# Reasoning has become a luxury

**Published:** 2014-04-08

**Authors:** G. Barbujani

**Affiliations:** Dipartimento di Biologia ed Evoluzione, Università di Ferrara (g.barbujani@unife.it)

**Keywords:** quality, research, journal

It is being talked about all over the world; an editorial in the Economist of 19 October 2013 entitled “How Science Goes Wrong” reports a dramatic decline in the quality of scientific publications. Research has changed the world, now it needs to change itself, the subheading reads.

The detailed report is based on facts which are difficult to disprove; an example being a Harvard biologist, John Bohannon, who sent an invented article full of nonsense on how to combat cancer using lichens to 304 scientific journals. Of this number, 157 accepted it for publication. While these publications were second tier, the problem affects them all, even those of greater importance.

“Publish or perish” is the rule and not just from today. However, today, researchers are forced to publish more and more in order to keep ahead of the competition, and to demonstrate by weight of their reputation that they deserve indispensible funding, in short, in order to survive. The bar has been raised therefore, says the “Economist”, causing a collapse in the control mechanisms tied to the reproducibility of the experiments and to those anonymous referees who have the task of judging the articles prior to submission to the journals.

This reproducibility, from Galileo onwards, is the cornerstone of the scientific method: the results of an experiment, independently conducted, must always be the same. If the anopheles mosquito really transmits malaria, whoever repeats the experiment will confirm that. However, this becomes an abstract idea when the cost of such projects are measured in millions of euros. Who will take the trouble to spend such an amount only to prove something that we already think we know?

The referees, who are supposed to prove the articles, are also scientists, and are also under pressure in their fight for survival where the first victim is the time necessary to work properly. Some of them still try to, some don’t, and thus experiments conducted in a haphazard fashion pass quality control unscathed, and statistically inconsistent results end up by being accepted as valid.

In part, this depends on the growing number of researchers. Darwin thought for 23 years prior to publishing “The Origin of Species”, but today, with 6 or 7 million scientists in the world, a week’s delay can make the difference between success and failure. Speed has become a necessity and reasoning a luxury. And in part, it is caused by the fact that the principal scientific journals, acquired by the large publishing groups, today have to produce a profit. If their articles are taken up by the newspapers and television, their reputation increases, and with it so does the revenue from advertising. Therefore, in many cases, the submitted articles initially pass through the hands of publicity experts, normally young people with almost no knowledge of science, but who return good articles to the senders if they think that they will not attract the attention of the media. In this way, publication in the most important scientific journals has increasingly become something of a lottery; you place your bet, hoping to draw the lucky number.

Gabriele Romagnoli wrote in “Repubblica” that football has had problems since the players started wearing boots in a variety of ridiculous colours. Something similar also happened in science. The habit has spread of inserting catch phrases with no relevance to the research but everything to do with its promotion. If all goes well these phrases are then taken up by the headline-writers of the newspapers and by the newly-created but already voracious press offices. A symptom, undoubtedly, not a cause, but it should be taken seriously. As in other sectors, science also has relied on the values of the market, hoping that it would self-regulate. But in what has become the science market, properly doing one project at a time sometimes does not pay, better to do a hundred; maybe ninety will be rubbish but the others will keep us afloat. Thus, the grand figure of the patient and critical research worker, who submits his results for verification after verification until he is thoroughly convinced, is now nonexistent. Nowadays, the world of research is dominated by entrepreneurial scientists, quick-thinking, clever at fund-raising, very much at their ease with the job of creating alliances, but less with that of critically evaluating to what extent a result holds up.

There are also those who try to bring a bit of rationality into the system. Jaume Bertranpetit, the director of Icrea, the Catalan equivalent of our National Research Council, selects its candidates on the basis of five articles: “I want people who, in their career, have completed five really good things. I am not interested in the rest”. At the Institute for Advanced Studies at Princeton they are even more selective; the articles to be presented in order to apply to spend a year in the institute which housed the exiled Albert Einstein are three in all. If this approach were to become widespread, many would be persuaded to back quality over quantity.

As stated at the beginning of this article, all of this is being discussed throughout the world, also because the future of advanced and very advanced productive sectors, and therefore millions of jobs, depend on the decisions taken. It is talked about much less in Italy where problems have doubled because of lack of funds and tripled because of the bureaucratic dictatorship, which paralizes the best researchers with periodic lethal injections of nonsense (the last of which being the restructuring of the research doctorate). Science has changed the world but here we haven’t noticed. It is clear that we prefer to debate the Stem Cell method or how retroactive the Severino Act is, and that’s the way the cookie crumbles! However, nobody should be surprised when, in a short time, we realize for the umpteenth time that the others make progress while we flounder.

The “Economist” [1] has reported a dramatic drop in the quality of scientific publications. An unbridled competition has caused the collapse of the control mechanisms.
